# Nrf2-dependent effects of CDDO-Me on bactericidal activity in macrophage infection models

**DOI:** 10.3389/fimmu.2025.1574776

**Published:** 2025-05-01

**Authors:** Therese B. Deramaudt, Ahmad Chehaitly, Marcel Bonay

**Affiliations:** ^1^ U1179 INSERM, END-ICAP, UFR des Sciences de la Santé-Simone Veil, Université de Versailles Saint-Quentin-en-Yvelines, Montigny-le-Bretonneux, France; ^2^ Service de Physiologie-Explorations Fonctionnelles bi-sites; Hôpitaux Ambroise Paré et Bicêtre, Boulogne-Billancourt, Le Kremlin-Bicêtre, France

**Keywords:** CDDO-Me, macrophages, inflammation, bronchoalveolar lavage, oxidative stress, bactericidal activity, *Staphylococcus aureus*, Nrf2 knockout mice

## Abstract

**Introduction:**

Diabetes and chronic kidney disease (CKD) increase susceptibility to bacterial infections, particularly *Staphylococcus aureus*, which is associated with highmortality in CKD patients. Dysregulated macrophage activity and excessive oxidative stress exacerbate immune dysfunction and inflammation in these conditions. Nrf2 (nuclear factor erythroid 2–related factor 2) is a key regulator of antioxidant defenses and macrophage function. CDDO-Me, a synthetic triterpenoid, activates Nrf2, providing antioxidant and anti-inflammatoryeffects. However, its precise role in modulating macrophage activity, polarization, and bacterial clearance remains unclear.

**Methods:**

The effects of CDDO-Me on macrophage function were evaluated *in vitro* (THP-1 and RAW 264.7 macrophages) and an *in vivo* Nrf2 knockout mouse model. Nrf2 activation was assessed via Western blot and luciferase reporter assays, oxidative stress was measured using CellROX reagent, and inflammatory responses were quantified by RT-qPCR. Intracellular *S. aureus* survival and macrophage polarization markers were analyzed to investigate the role of CDDO-Me in enhancing bactericidal activity.

**Results:**

Our results showed that CDDO-Me activated the Nrf2 signaling pathway, reducing oxidative stress and inflammation in macrophages by downregulating pro-inflammatory cytokines (IL-1β, TNF-α). It modulated macrophage polarization, decreasing M1 and M2 marker expression, and significantly enhanced bactericidal activity against *S. aureus*. These effects were Nrf2-dependent, as demonstrated in knockout models.

**Conclusion:**

The ability of CDDO-Me to regulate oxidative stress, inflammation, and bacterial clearance underscores its therapeutic potential for managing inflammatory and infectious diseases indiabetes and CKD.

## Introduction

People with diabetes are at an increased risk of bacterial infections, such as foot infections and urinary tract infections, often caused by high sugar and multidrug-resistant bacteria (1).This vulnerability is further exacerbated in individuals with chronic kidney disease (CKD), as CKD weakens the immune system and impairs the body’s ability to fight infections. Among the various pathogens, *Staphylococcus aureus* poses a significant threat. Studies on hemodialysis-dependent patients indicate that infections frequently arise from the patients’ own *S. aureus* carriage isolates (2). Even in non-dialysis-dependent CKD patients, *S. aureus* bacteremia is associated with markedly increased mortality, highlighting the severe impact of infection in this population (3). Moreover, oxidative stress appears to be an important contributor to the progression of diabetic kidney disease (4, 5).

Inflammation serves as a critical early defense mechanism of the innate immune system, targeting pathogens such as bacteria and viruses, while promoting tissue repair and restoring homeostasis (6). A crucial feature of inflammation is the production of reactive oxygen species (ROS) and nitrogen oxygen species, which are highly toxic to pathogens and prevent their tissue invasion. A hallmark of inflammation is the recruitment of immune cells, such as macrophages and lymphocytes, and the synthesis of signaling molecules, including cytokines and chemokines, which coordinate the immune response (7).

Macrophages are major players in the first line of defense to bacterial infections. They serve a dual role in maintaining immune homeostasis under normal conditions and orchestrating robust immune responses during infections and inflammation. Macrophages exhibit remarkable plasticity, allowing them to adopt distinct functional phenotypes depending on their surrounding environment. These phenotypes range from the pro-inflammatory, classically activated M1 phenotype, essential for microbial killing and initiating immune responses, to the anti-inflammatory, alternatively activated M2 phenotype, which facilitates tissue repair and resolution of inflammation (8). However, dysregulation of macrophage activity or polarization contributes to pathological conditions, including chronic inflammatory diseases and impaired immune responses to infections (8–10). Prolonged oxidative stress and excessive production of ROS can further exacerbate tissue damage and hinder immune function (11).

The transcription factor Nrf2 (nuclear factor erythroid 2–related factor 2) plays a vital role in mitigating oxidative stress and regulating macrophage function (12). Under homeostatic conditions, Nrf2 is sequestered in the cytoplasm by its inhibitor, Keap-1, which targets it for degradation (13, 14). However, during cellular stress, Nrf2 dissociates from Keap-1, translocates to the nucleus, activating antioxidant response elements (ARE) to control the expression of genes involved in detoxification, antioxidation, cytoprotection, and anti-inflammation (15, 16). Activating the Nrf2 signaling pathway is a promising therapeutic strategy for inflammatory and oxidative stress-related diseases, including diabetic complications (17, 18).

CDDO-Me (Methyl-2-cyano-3,12-dioxooleana-1,9(11)-dien-28-oate), a synthetic triterpenoid derived from oleanolic acid, has gained significant attention due to its ability to activate Nrf2 and exert potent antioxidant, anti-inflammatory, and cytoprotective effects in various cellular and disease models (19–22). CDDO-Me interacts with Keap1, an Nrf2 inhibitor, and modifies its Cys151 residue, disrupting its binding to Cullin 3 and allowing Nrf2 accumulation (23).

CDDO-Me has shown efficacy in various diseases, including diabetic nephropathy, cancer (leukemia, solid tumors), and neurological disorders (4, 19, 24–26) through the modulation of signaling pathways such as Nrf2, NF-κB, PI3K/AKT/mTOR, MAPK, and JAK/STAT pathways (27–30). A Japanese phase II clinical trial demonstrated that CDDO-Me improved renal function in patients with CKD associated with type 2 diabetes by enhancing glomerular filtration rate (31).

Despite its promising therapeutic potential, the precise mechanisms through which CDDO-Me modulates macrophage function and influences polarization, oxidative stress, and bacterial activity, remain unclear. To investigate this, we evaluated the effects of CDDO-Me *in vitro* using THP-1-derived and RAW 264.7 macrophages and an *in vivo* Nrf2 knockout mouse model. Our findings reveal how CDDO-Me influences cell viability, oxidative stress, inflammatory cytokine expression, macrophage polarization, and bactericidal activity, offering new insights into its role as a multifunctional therapeutic agent for inflammatory and infectious diseases.

## Materials and methods

### Antibodies, reagents, and compounds

Phorbol 12-myristate 13-acetate (PMA), human interferon gamma (IFNγ), lipopolysaccharide (LPS), dimethyl sulfoxide (DMSO), anti-GAPDH antibody, β-mercaptoethanol, and bardoxolone methyl (CDDO-Me) were purchased from Sigma (Merck). Dual Luciferase (Firefly-Renilla) Assay System was purchased from BPS Bioscience. DMEM (Dulbecco’s Modified Eagle Medium), RPMI (Roswell Park Memorial Institute) 1640 culture medium, phosphate-buffered saline (PBS), and heat-inactivated fetal bovine serum were obtained from Eurobio Scientific. MTT cell viability assay kit (Biotium), Tryptic soy broth and tryptic soy agar (Conda laboratories) were purchased from Dutscher. Live/Dead™ BacLight™ bacterial viability kit, CellROX™ Green Oxidative Stress Reagent, iBind Western blot system, RIPA Lysis and Extraction Buffer, Pierce protease and phosphatase inhibitor cocktail, Maxima First Strand cDNA synthesis kit, Fluoromount-G™ (EMS), Lipofectamine^®^ LTX & PLUS™ Reagent, and Trizol were purchased from ThermoFisher scientific. DC Protein Assay Reagents, 4-20% mini-Protean precast protein gels, and iTaq SYBR green supermix were purchased from Bio-Rad. Anti-Lamin AC antibody, anti-HO-1, and gentamicin were purchased from Abcam. Anti-Nrf2 antibody was obtained from ProteinTech. IRDye^®^ 680CW goat anti-mouse IgG, and IRDye^®^ 800CW Goat anti-Rabbit IgG secondary antibodies were purchased from LI-COR^®^ Biosciences.

### Animals and isolation of primary mouse bronchoalveolar macrophages

C57BL/6J Nrf2 knockout (*Nrf2^-/-^*) mice, provided by Dr. Yamamoto (Tohoku University, Japan) and purchased from Riken BRC (32), were bred at the UFR des Sciences de la Santé Simone Veil-Université de Versailles Saint-Quentin-en-Yvelines under the license APAFIS#28944-2021010815458509v3 approved by the Institutional Animal Care and Use Committee, and the Ministère de l’Enseignement supérieur de la Recherche et de l’Innovation. Nrf2 heterozygote (*Nrf2^+/-^*) littermates were used as control to *Nrf2^-/-^* mice. Mice were maintained in a standard 12 h light/12 h dark cycle with access to food and water *ad libitum*.

Isolation of bronchoalveolar macrophages was performed via bronchoalveolar lavage (BAL). BAL was conducted following carbon dioxide euthanasia of mice, in accordance with ethical standards. Alveolar macrophages were collected through repeated lavages with 1 mL PBS. For each experimental condition, BAL fluid from a pool of 3 mice was required to obtain a sufficient number of bronchoalveolar macrophages. After cell resuspension, total cell counts were determined using the Countess automated cell counter (Invitrogen, ThermoFisher Scientific),. Primary alveolar macrophages were maintained overnight in complete DMEM supplemented with a penicillin/streptomycin mixture. The antibiotics were subsequently removed by washing with PBS prior to performing *ex vivo* experiments, followed by suspension in antibiotics-free medium.

### Cell culture, CDDO-Me preparation, and cell viability assay

RAW 264.7 macrophages (ATCC^®^ TIB-71™) were maintained in DMEM supplemented with 10% heat-inactivated fetal bovine serum, 1 mM sodium pyruvate, in a humidified atmosphere at 37°C and 5% CO_2_. Human monocytic THP-1 cell line (ATCC^®^ TIB-202™) was maintained in RPMI 1640 Glutabio medium supplemented with 10% heat-inactivated fetal bovine serum, 10 mM HEPES buffer, 1 mM sodium pyruvate, and 50 µM β-mercaptoethanol in a humidified atmosphere at 37°C and 5% CO_2_. Terminal differentiation of THP-1 to macrophages was obtained by washing cells twice with PBS prior to incubation with 50 nM PMA in β-mercaptoethanol-free complete RPMI 1640 medium for 48 h. After differentiation, cells were rinsed twice with PBS to remove PMA and fresh complete medium was added.

For *in vitro* experiments, CDDO-Me was initially resuspended in DMSO at 15 mM then diluted in PBS to the indicated concentrations. For *in vivo* studies, CDDO-Me was administered via intraperitoneal injection at a dose of 2 mg/kg (33). The control group corresponds to DMSO diluted in PBS.

Cell viability was assessed using the MTT assay. THP-1-derived macrophages were seeded at 5 x 10^4^ cells/well in a 96-well plate. Cytotoxicity to control, 30% ethanol, or indicated concentrations of CDDO-Me was assessed 24 h after treatment by using 3-(4,5-dimethylthiazol-2-yl)-2,5-diphenyltetrazolium bromide (MTT) cell viability assay kit to measure cellular metabolic activity following the manufacturer’s instructions. Absorbance changes were measured at 550 nm, with background absorbance measured at 600 nm, using the FLUOstar Omega microplate reader (BMG Labtech).

### LPS stimulation and LPS/IFNγ-mediated M1 polarization of macrophages

For LPS stimulation, THP-1-derived macrophages cultured in 6-well plates at 1 × 10^6^ cells/well were pretreated with control or CDDO-Me (1, 5, 10, 25, 50 nM). After 3 h, low-dose LPS (5 ng/ml) was added to the medium. After 3 h of additional incubation, total RNAs were extracted for analysis.

For M1 polarization, 24 h after PMA treatment, THP-1-derived macrophages were washed twice with PBS, then fresh culture medium supplemented with 100 ng/ml LPS and 20 ng/ml IFNγ were added for 48 h incubation to obtain proinflammatory macrophages (M1). After medium change, M0 and M1 macrophages were treated with control or CDDO-Me for an additional 48 h incubation.

### Subcellular fractionation and immunoblotting

For total protein extraction, 1 × 10^6^ cells/well were treated with control or CDDO-Me for 24 h before being washed twice with cold PBS and lysed with ice-cold RIPA buffer supplemented with a cocktail of protease inhibitors. After 30 min on ice, cell lysates were centrifuged at 12,000 x g and supernatants containing the protein extracts were collected.

For nuclear fractionation, THP-1-derived macrophages at 3 × 10^6^ cells/25-cm^2^ flask were treated with control or CDDO-Me for 8 h before protein extraction. Briefly, macrophages were washed twice with PBS and lysed with buffer A (10 mM HEPES, 1.5 mM MgCl_2_, 10 mM KCl, 0.5 mM DTT, 0.05% NP40, pH 7.9) supplemented with a cocktail of protease inhibitors. After 10 min incubation on ice, the cell lysates were centrifuged at 900 x g. The cell pellets containing the nuclear fractions were resuspended in buffer B (5 mM HEPES, 1.5 mM MgCl_2_, 0.2 mM EDTA, 0.5 mM DTT, 26% glycerol, pH 7.9) supplemented with a cocktail of protease inhibitors. After 30 min on ice, followed by centrifugation at 10,000 x g, the supernatants containing the nuclear fractions were collected.

Protein concentrations were quantified using the DC protein assay kit (Bio-Rad) and protein extracts were resolved by SDS-PAGE on 4-20% gradient gels. After protein transfer on polyvinylidene difluoride membranes (Immobilon-FL, Merck), western blots were performed using the iBind Flex western system (Invitrogen, ThermoFisher scientific) following the manufacturer’s instructions. Briefly, primary antibodies targeting Nrf2, HO-1, GAPDH, lamin AC, and secondary antibodies, IRDye680RD and IRDye800RD, were diluted in iBind Flex FD solution. Fluorescence signals were acquired using Odyssey CLx imaging system (LI-COR) and densitometric analysis was achieved using Image Studio Lite v4.0.

### Total RNA isolation, reverse transcription, and real-time quantitative PCR analysis

Total RNA was isolated from macrophages treated with either control or CDDO-Me using the Trizol reagent and chloroform extraction technique following the manufacturer’s instructions. RNA concentrations were determined using the NanoPhotometer^®^ N120 (Implen; München, Germany). One µg of total RNA was reverse transcribed to cDNA using Maxima First strand cDNA synthesis kit. Quantitative analysis was achieved using real-time quantitative PCR (RT-qPCR), with each cDNA sample done in triplicate. qPCR was realized using the Bio-Rad CFX384 Touch Real-Time PCR Detection system (Bio-Rad) and iTaq SYBRgreen qPCR mix. **Table 1** lists the specific primers used for qPCR, IL-6, IL-1β, TNF-α, CCR7, IL-23, PPARγ, CCL22, IL-10, and 18S rRNA, and synthetized by Eurogentec. The cycle threshold (Ct) values of each target genes were first normalized to that of the reference gene 18S rRNA (ΔCt) then the final values (ΔΔCt values) were expressed as folds of control. Data were analyzed on the Bio-Rad CFX manager v3.1 using the ΔΔCt method.

**Table 1:** Primers used in qPCR for THP-1-derived macrophages.

Name	Forward primer sequences (5'-3')	Reverse primer sequences (5'-3')
Human IL-23	GTTCCCATACCAGTGTGG	GAGGCTTGGAATCTGCTGAG
Human CCR7	GATTACATCGGAGACAACACCA	AGTACATGATAGGGAGGAACCAG
Human IL-1β	AATGATGGCTTATTACAGTGGCA	GTCGGAGATTCGTAGCTGGA
Human IL-6	GTAGCCGCCCCACACAGA	CATGTCTCCTTTCTCAGGGCTG
Human TNF-α	GGAGAAGGGTGACCGACTC	TGGGAAGGTTGGATGTTCGT
Human PPARγ	TTCAGAAATGCCTTGCAGTG	CCAACAGCTTCTCCTTCTCG
Human CCL22	ATTACGTCCGTTACCGTCTG	TAGGCTCTTCATTGGCTCAG
Human IL-10	TCAAGGCGCATGTGAACTCC	GATGTCAAACTCACTCATGGCT
Human 18S rRNA	GATAGCTCTTTCTCGATTCCG	CTAGTTAGCATGCCAGAGTC

### Nrf2 activity assay

Nrf2 activity was quantified using ARE-Reporter kit (BPS Bioscience). Briefly, 7.5 × 10^4^ THP-1 cells per well were seeded in 96-well plates and differentiated with PMA. After 24 h incubation, THP-1-derived macrophages were transfected with ARE-Firefly luciferase reporter vector mixed with constitutively-expressing Renilla luciferase reporter vector using the Lipofectamine^®^ LTX & PLUS™ Reagent (ThermoFisher scientific) following the manufacturer’s instructions, and incubated for 24 h. Transfected THP-1-derived macrophages were treated with control or CDDO-Me for an additional 48 h. Chemiluminescence signal from the dual luciferase Firefly-Renilla assay system was measured on the FLUOstar Omega microplate reader (BMG Labtech). Nrf2 activity resulted from the ratio of firefly luminescence signal to the corresponding Renilla luminescence signal.

### Quantification of intracellular ROS production

ROS production was measured using the CellROX™ Green Oxidative Stress kit (ThermoFisher scientific). Briefly, 2.5 × 10^5^ cells/well of THP-1 were seeded on coverslips placed in 24-well plates and cells were differentiated with PMA for 48 h. Briefly, macrophages were treated with control or 25 nM CDDO-Me for 3 h prior to addition or not of 5 ng/ml LPS followed by an additional 4 h incubation. Live macrophages were stained with CellROX reagent for 30 min, washed with PBS and fixed with 4% paraformaldehyde solution. Nuclei were counterstained with 4′, 6-diamidino-2-phenylindole (DAPI), and cells were mounted with the Fluoromount-G aqueous mounting medium. Fluorescent images were taken with Leica SP8 confocal microscope (Leica Microsystems) and fluorescent signals were analyzed from selected 7 fields per treatment using Image J v1.53k (National Institutes of Health).

### Bacteria strains and growth culture

The gram-positive bacteria, *Staphylococcus aureus* strain (ATCC 25923), was grown aerobically in Trypticase soy broth to the optical density of 1 at 37°C under agitation. Bacterial glycerol stocks were prepared and when required, frozen stocks were thawed and bacteria were diluted in PBS at the appropriate MOI. For intracellular bacterial survival assay, bacteria were seeded on Trypticase soy broth solidified with 1.5% agar.

### Bacteria intracellular survival assay

THP-1-derived macrophages and bronchoalveolar macrophages, seeded in 24-well plates at 2.5 × 10^5^ cells/well, were treated with control or 25 nM CDDO-Me for 24 h prior to infection with *S. aureus* at an MOI of 10. Gentamycin was added to the cell culture medium 1 h after infection to kill any extracellular bacteria, and cells were incubated for an additional 24 h before colony forming unit (CFU) assay. Briefly, cells were washed twice with PBS and lysed cells in 1 ml ice-cold sterile water for 20 min. Numeration of intracellular bacteria was obtained by plating 5-fold serial dilutions on Trypticase soy agar plates and incubating at 37°C for 24 h.

### Bacteria viability

Viability of *S. aureus* was assessed using the Live/Dead™ BacLight™ bacterial viability kit (ThermoFisher scientific) according to the manufacturer’s recommendations. SYTO9 was used to stain bacteria and Propidium Iodide (PI) to stain membrane-damaged bacteria. Stained bacteria were incubated with control or 25 nM CDDO-Me for 90 min. During that incubation time, fluorescent signals were monitored on the FLUOstar Omega microplate reader (BMG Labtech) at 45, 60, and 90 min using 488 nm excitation and measuring emission signals at 530 nm and 630 nm.

### Statistical analysis

Data are expressed as mean ± standard errors of the mean (SEM). All experiments were performed independently and derived from at least 3 independent replicates. Statistical comparisons and graph design were conducted using GraphPad Prism (v8). Student’s unpaired *t*-test was used for comparison between two groups, while one-way ANOVA was used for comparisons among multiple groups. A p value <0.05 was considered significant.

## Results

### CDDO-Me activates the Nrf2 signaling pathway

**Figure 1:**
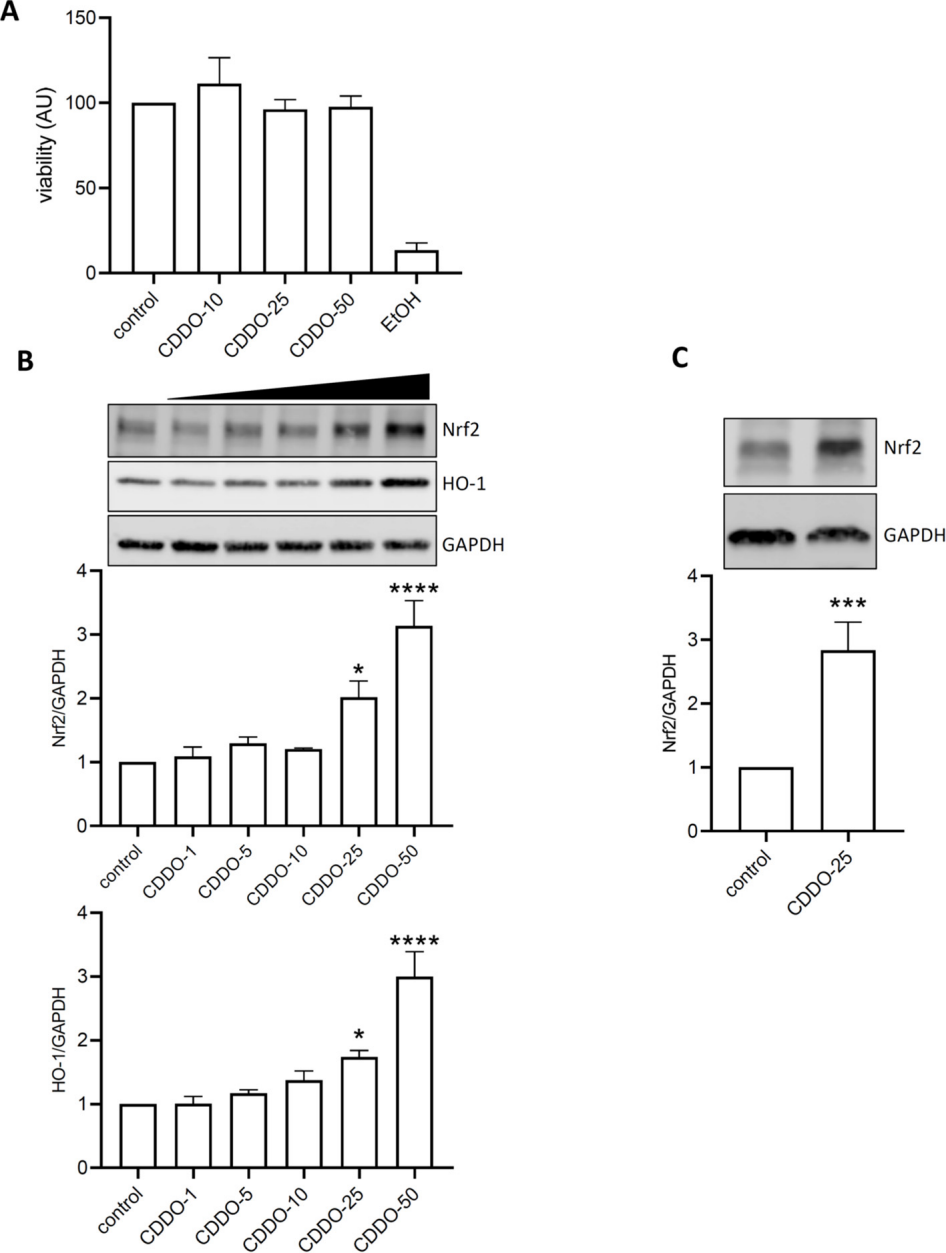
CDDO-Me activated Nrf2 signaling pathway. **(A)** THP-1-derived macrophages were treated with 10, 25, or 50 nM CDDO-Me for 24 h before testing for cell viability using MTT assay (n=5). Positive control for cell death was obtained by incubating cells with EtOH. **(B)** Raw 264.7 macrophages were incubated for 24 h in presence of the indicated concentrations of CDDO-Me. Proteins from whole cell lysates were analyzed by Western blot and Nrf2 protein expression levels were detected using specific antibodies for Nrf2 (n=4), HO-1 (n=5), and GAPDH. Immunoblots are representative of 4 independent experiments, Data are presented as mean ± SEM and comparisons were done using one-way ANOVA. **(C)** THP-1-derived macrophages were treated with 25 nM CDDO-Me. Images are representative of 5 independent experiments, Student’s t-test. *p<0.05, **p<0.005, and ****p<0.001.

The toxicity of CDDO-Me was initially tested in THP-1-derived macrophages at concentrations of 10, 25, and 50 nM. Cells were incubated with CDDO-Me for 24 h, and viability was assessed using an MTT assay (**Figure 1A**). Incubation with 5% ethanol was used as positive control. The results showed no significant cell death at any concentration compared to the control cells. In RAW 264.7 macrophages, CDDO-Me activated the Nrf2 signaling pathway (**Figure 1B**). Specifically, treatment with 25 nM CDDO-Me significantly increased the expression of Nrf2 and HO-1 proteins.

Similarly, THP-1-derived macrophages responded to 25 nM CDDO-Me stimulation by showing an increase in Nrf2 protein expression (**Figure 1C**).

### CDDO-Me induces Nrf2 nuclear translocation and Nrf2 activity

**Figure 2:**
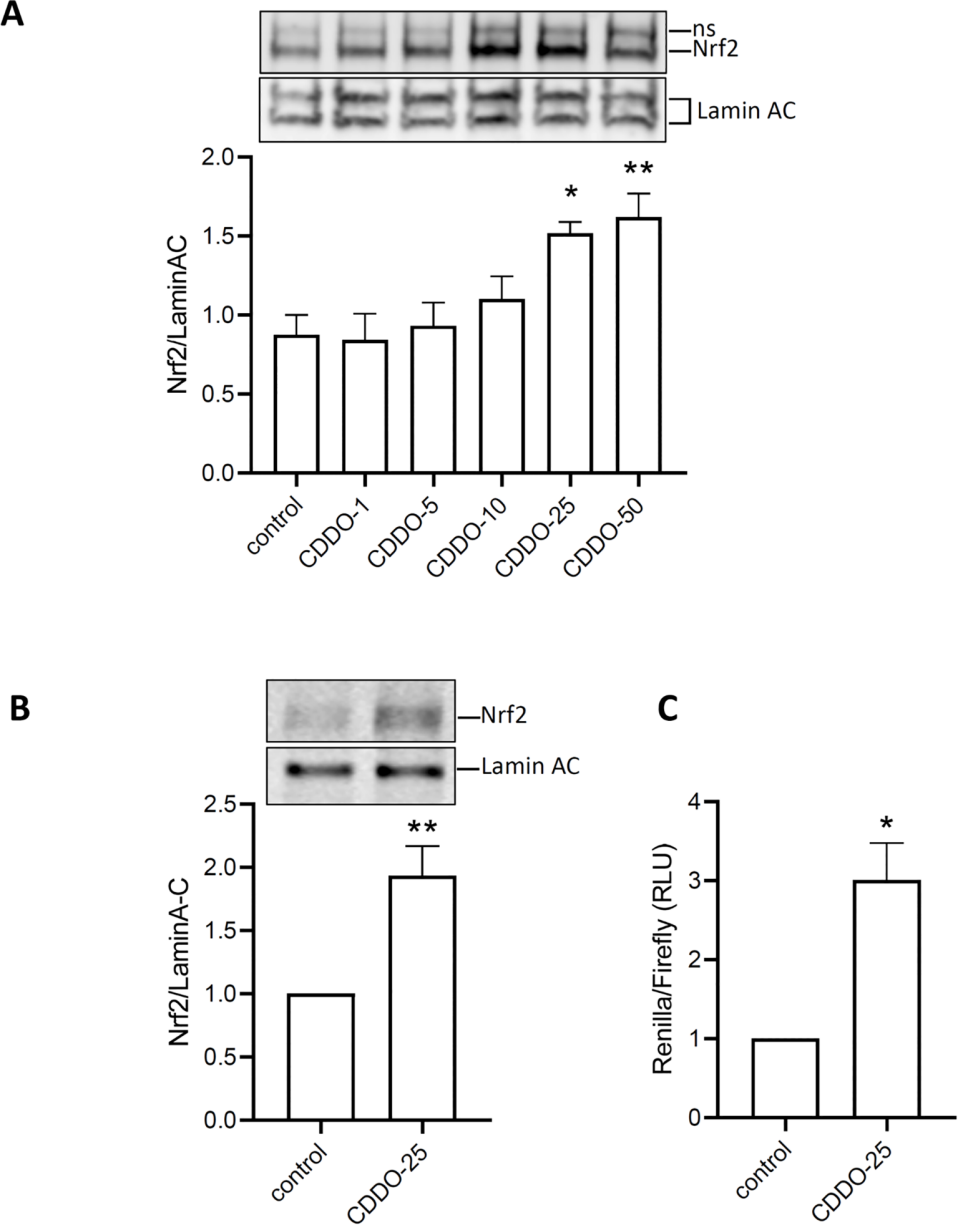
Activation of Nrf2 by CDDO-Me. **(A)** nuclear translocation of Nrf2 was observed in protein extracted 6 h after RAW 264.7 was incubated with increasing concentrations of CDDO-Me (n=5; one-way ANOVA). ns: non-specific signal. **(B)** Nuclear translocation of Nrf2 was also observed in THP-1-derived macrophages treated with CDDO-Me 25 nM (n=5, Student’s t-test). **(C)** THP-1-derived macrophages, transfected with ARE-Luciferase vector for 24 h, were treated with CDDO-Me 25 nM for an additional 24 **(h)** Expression of ARE-Luciferase reporter was assessed by chemiluminescent assay (n=3 independent experiments done in triplicate, Student’s t-test). *p<0.05 and **p<0.01.

To determine whether CDDO-Me activates Nrf2, RAW 264.7 macrophages were treated with increasing concentrations of CDDO-Me for 24 h. Nuclear extracts were collected and analyzed by Western blot (**Figure 2A**). The data showed that CDDO-Me began to stimulate Nrf2 translocation into the nucleus at 10 nM concentration and significantly induced Nrf2 translocation at concentrations of 25 nM and 50 nM. Based on its lack of effect on cell viability and its ability to induce Nrf2 nuclear translocation, 25 nM was selected as the optimal concentration of CDDO-Me for further experiments.

When THP-1-derived macrophages were treated with CDDO-Me, nuclear extracts analysis by Western blot also revealed significant Nrf2 nuclear translocation (**Figure 2B**). To assess Nrf2 activity, THP-1-derived macrophages were transfected with ARE-reporter, and Firefly-Renilla luciferase assays were performed. The results showed a significant increase in luminescence in CDDO-Me-treated macrophages compared to control macrophages (**Figure 2C**). These findings suggest that 25 nM CDDO-Me stimulates Nrf2 nuclear translocation and activates the Nrf2 signaling pathway.

### Antioxidant effect of CDDO-Me on LPS-stimulated macrophages

**Figure 3:**
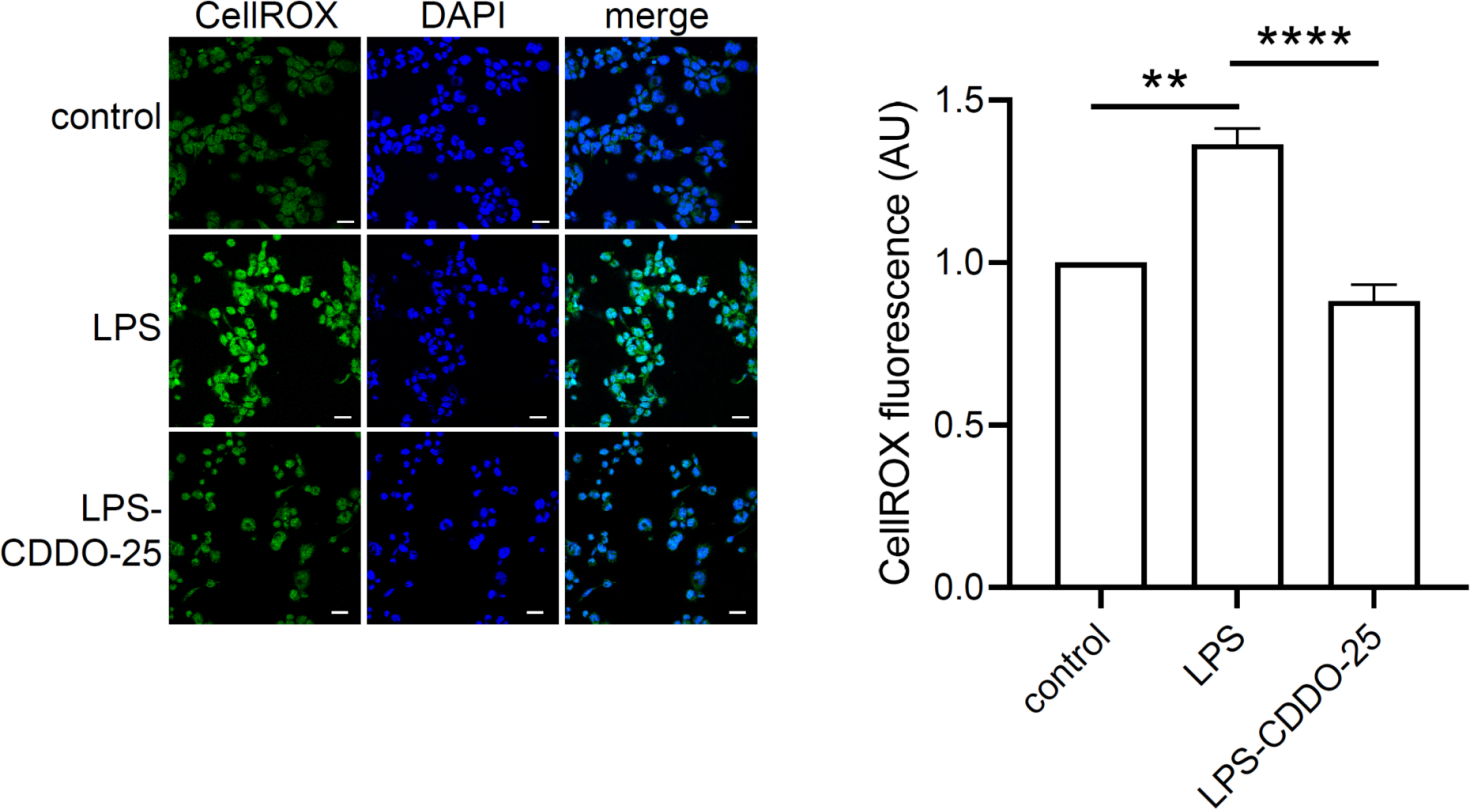
Antioxidant effect of CDDO-Me on LPS-stimulated THP-1-derived macrophages. THP-1-derived macrophages were treated with 25 nM CDDO-Me for 3 h prior to stimulation with 5 ng/ml LPS. After 3 h, live cells were stained with CellROX reagent. Stained cells were PFA-fixed, and nuclei counterstained with DAPI. Seven images were taken by confocal microscopy and CellROX fluorescent signals were analyzed using Image J software (n=3 independent experiments; Scale bar = 20 µm). **p<0.01 and ****p<0.001 using one-way ANOVA.

To determine whether CDDO-Me activation of Nrf2 impacts oxidative stress in macrophages, THP-1-derived macrophages were treated with CDDO-Me for 3 h prior to stimulation with 5 ng/ml LPS, followed by an additional 3 h incubation. CellROX reagent was added to live cells to quantify of oxidative stress (**Figure 3**). Macrophages were fixed with 4% PFA, counterstained with DAPI, and imaged using a confocal microscopy.

The data showed a significant increase in ROS levels in LPS-stimulated THP-1-derived macrophages. However, pretreatment with CDDO-Me significantly reduced LPS-induced ROS levels compared to macrophages stimulated with LPS alone. These results suggest that CDDO-Me treatment efficiently suppresses LPS-induced oxidative stress in macrophages.

### Anti-inflammatory effect of CDDO-Me in THP-1-derived macrophages

**Figure 4:**
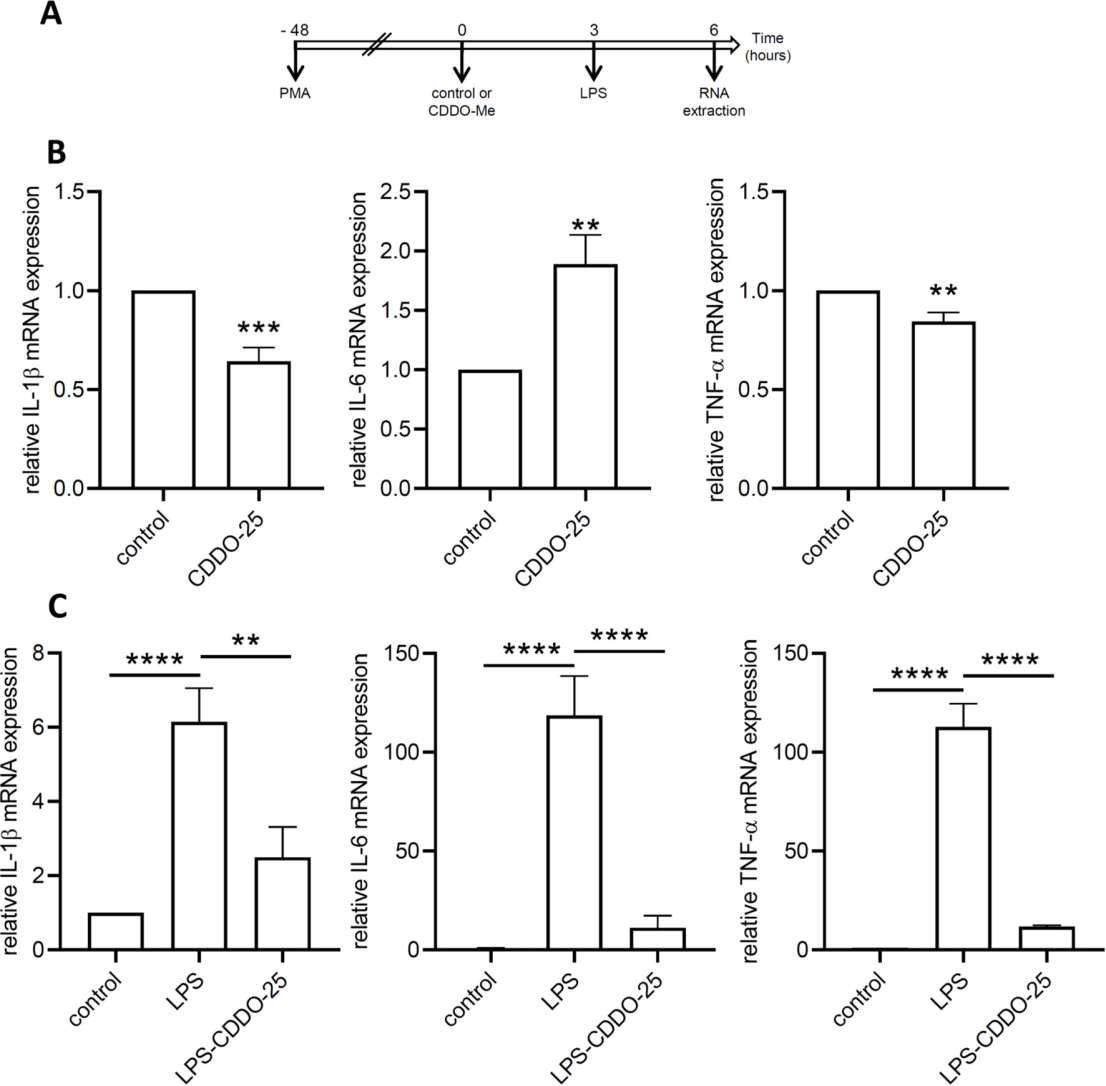
Anti-inflammatory effect of CDDO-Me. **(A)** Experimental design. **(B)** THP-1-derived macrophages were treated with 25 nM CDDO-Me or control for 6 (h) Total RNA was extracted and genes coding for IL-1β, IL-6, and TNF-α were quantified by RT-qPCR (n=5). Data are presented as mean ± SEM. **(C)** THP-1-derived macrophages were pretreated with control or CDDO-Me prior to stimulation with low-dose LPS and incubation for an additional 3 (h) Genes coding for IL-1β, IL-6, and TNF-α were quantified by qPCR (n=5 independent experiments done in triplicate, one-way ANOVA). **p<0.01, ***p<0.005, and ****p<0.001. Analysis were performed by comparing two or three groups using Student’s t-test or one-way ANOVA, respectively.

We sought to examine the effect of CDDO-Me on inflammation using RT-qPCR. THP-1-derived macrophages were treated with CDDO-Me for 6 h, after which total RNA was collected (**Figure 4A**). The expression of inflammatory markers (IL-1β, IL-6, and TNF-γ) was assessed by qPCR.

In control THP-1 macrophages, CDDO-Me treatment resulted in a significant decrease in IL-1β and TNF-α, while IL-6 mRNA expression was increased (**Figure 4B**). In low-dose LPS-stimulated THP-1-derived macrophages, a significant increase in the expression of genes encoding IL-1β, IL-6, and TNF-α was observed (**Figure 4C**).

When macrophages were pretreated with CDDO-Me prior to LPS stimulation, the expression levels of all three genes (IL-1β, IL-6, TNF-α) were significantly downregulated compared to LPS-stimulated macrophages. These results demonstrate the anti-inflammatory effects of CDDO-Me on macrophages.

### CDDO-Me modulates the M1/M2 profile of THP-1-derived macrophages

**Figure 5:**
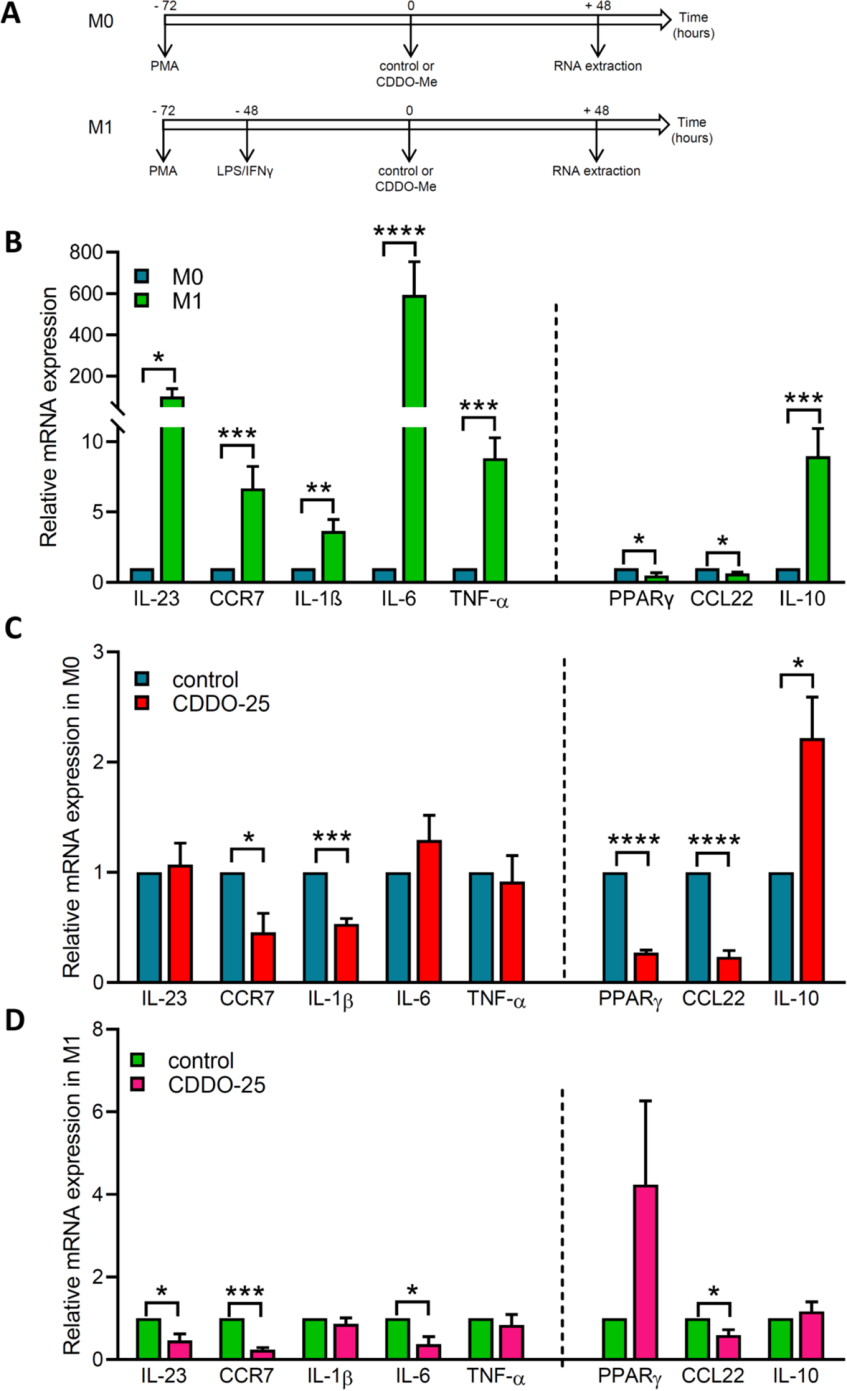
Effect of CDDO-Me on genes coding for M1/M2 polarization markers. **(A)** Experimental designs for M0 macrophages and M1 macrophages. M0 macrophages were obtained by differentiation of THP-1 cells into macrophages using PMA. M1 macrophages were obtained by incubating THP-1-derived macrophages with 100 ng/ml LPS and 20 ng/ml IFNγ. Control or 25 nM CDDO-Me were added to macrophages 72 h after PMA differentiation, and incubated for an additional 48 h before RNA extraction and RT-qPCR analysis. Graphs showed on the left side of the dash line M1 marker genes (IL-23, CCR7, IL-1β, IL-6, and TNF-α), and on the right side M2 marker genes (PPARγ, CCL22, and IL-10). **(B)** Fold-change mRNA expression levels in control-treated M1 macrophages and control-treated M0 macrophages. **(C)** Fold-change mRNA expression levels in control-treated M0 macrophages and CDDO-Me-treated M0 macrophages. **(D)** Fold-change mRNA expression levels in control-treated M1 macrophages and CDDO-Me-treated M1 macrophages (n=3 independent experiments done in triplicate). Student’s t-test was used to compare genes from CDDO-Me-treated macrophages and control-treated macrophages *p< 0.05, **p<0.01, ***p< 0.005, ****p< 0.0001.

Next, we examined the effect of CDDO-Me on macrophages phenotypically modified by treatment, specifically M0 and M1 macrophages. THP-1-derived macrophages were differentiated with PMA and identified as the M0 phenotype. M0 macrophages were then treated with IFNγ and LPS for 48 h to induce differentiation into M1 macrophages (**Figure 5A**). The modulation of gene expression for M1 markers (IL-1β, IL-6, TNF-α, IL-23, CCR7) and M2 markers (IL-10, PPARγ, CCL22) was examined by RT-qPCR.

The data showed increased expression of genes encoding M1 markers and the M2 marker IL-10 in M1 macrophages compared to M0 macrophages (**Figure 5B**). In contrast, the M2 markers PPARγ and CCL22 showed a significant decrease in expression.

In M0 macrophages treated with either control or CDDO-Me, a significant decrease in the expression of the M1 markers CCR7 and IL-1β was observed in CDDO-Me-treated cells compared to control-treated cells, while the expression of IL-23, IL-6, and TNF-α remained unchanged (**Figure 5C**). IL-10 expression was notably increased in macrophages treated with CDDO-Me compared to those treated with control.

In M1 macrophages treated with either control or CDDO-Me, the expression of IL-23, CCR7, IL-6, and CCL22 was significantly decreased, whereas the expression of IL-1β, TNF-α, PPARγ, and IL-10 was unchanged (**Figure 5D**).

These results suggest that CDDO-Me treatment reduces the M1 phenotype while having no significant effect on genes encoding M2 markers.

### CDDO-Me reduces intracellular survival of *S. aureus*

**Figure 6:**
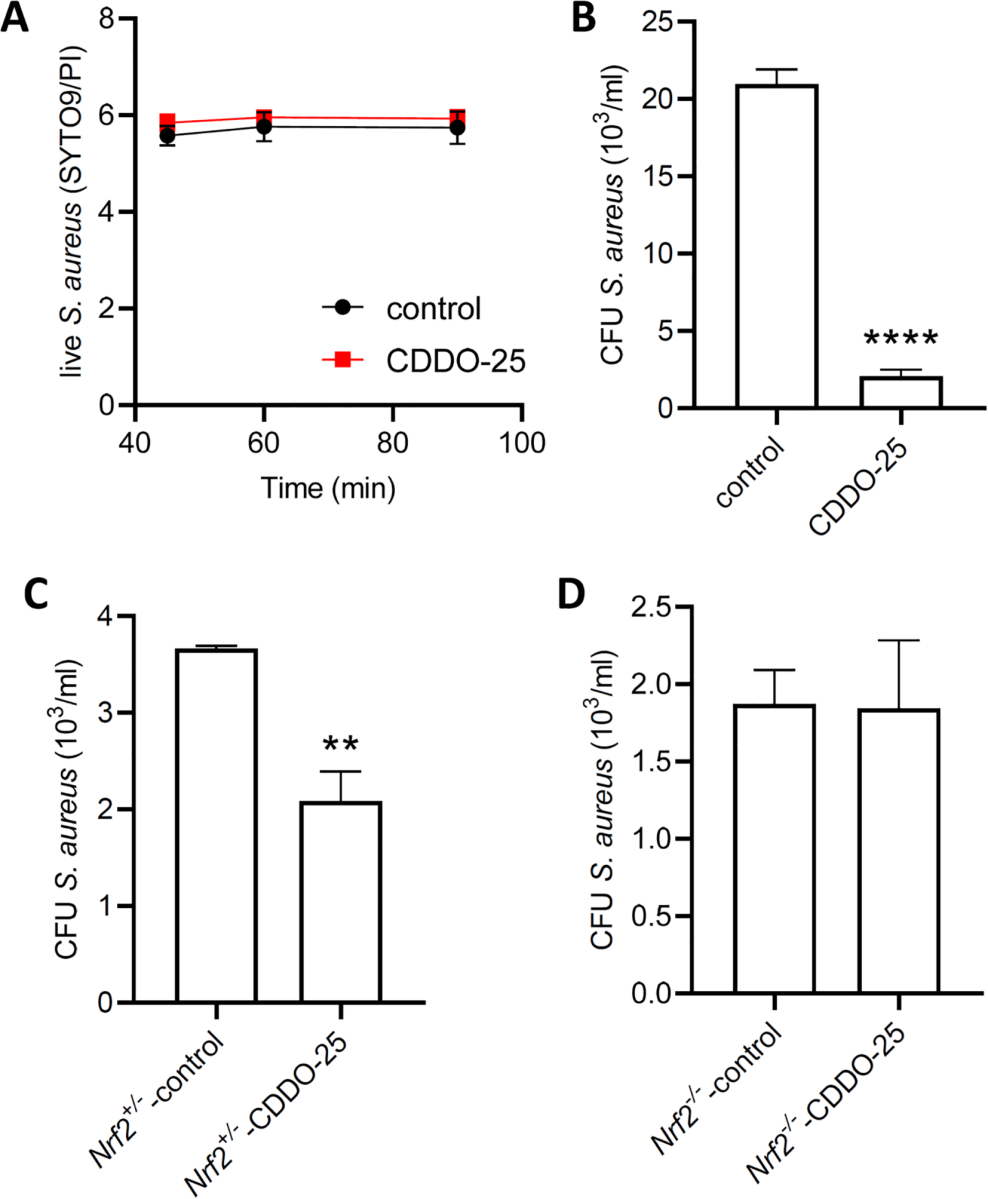
Effect of CDDO-Me on the bactericidal activity of macrophages. Bacterial viability was assessed using the BacLight bacterial viability kit. **(A)** *S. aureus* was incubated for 90 minutes in PBS containing 25 nM CDDO-Me or control. Fluorescent signals were measured at 45, 60, and 90 minutes by spectrophotometry (n=4 independent experiments done in triplicate). **(B)** THP-1-derived macrophages were pretreated with control or CDDO-Me for 24 h, infected for 1 h with *S. aureus* at MOI 10, before addition of gentamycin to the medium to eliminate extracellular bacteria. Intracellular survival of *S. aureus* was assessed 24 h after infection by CFU counts (n=4 independent experiments). **(C, D)** Macrophages isolated from BAL of *Nrf2^+/-^* control littermates **(C)** and *Nrf2^-/-^* mice **(D)** were submitted to the same treatment and infected with *S. aureus* at MOI 10. CFU counts of *S. aureus* was determined 24 h after infection (n=4 independent experiments). **p<0.01 and ****p<0.001.

To determine whether CDDO-Me affects macrophage activity, we performed an intracellular bacterial survival assay. First, we assessed the toxicity of CDDO-Me on *S. aureus* using the Live/Dead bacteria viability kit. Data were collected at 3 different time points using a spectrophotometer, which showed that *S. aureus* viability was not significantly affected by incubation with CDDO-Me (**Figure 6A**). Next, THP-1-derived macrophages were pretreated with control or CDDO-Me for 24 h before infection with *S. aureus*. One hour after infection, gentamicin was added to the medium to eliminate extracellular bacteria that had not penetrated the macrophages. Intracellular *S. aureus* survival was assessed 24 h post-infection using a colony-forming unit (CFU) assay. The results showed that CDDO-Me significantly decreased intracellular *S. aureus* survival compared to control macrophages (**Figure 6B**).

These effects observed in THP-1-derived macrophages were also detected in macrophages isolated from bronchoalveolar lavages collected from control *Nrf2^+/-^* mice intraperitoneally injected with control (**Figure 6C**). However, in *Nrf2^-/-^* mice injected with CDDO-Me, there was no change in bactericidal activity compared to control-injected *Nrf2^-/-^* mice (**Figure 6D**). These findings suggest that the increase in macrophage bactericidal activity following CDDO-Me treatment is Nrf2-dependent.

## Discussion

This study examined the multifaceted roles of CDDO-Me in macrophage modulation, particularly its effects on the Nrf2 signaling pathway, oxidative stress, inflammation, macrophage polarization, and bactericidal activity.

Our findings demonstrate that CDDO-Me is a potent activator of the Nrf2 signaling pathway in RAW 264.7 and THP-1-derived macrophages. Treatment with 25 nM CDDO-Me activates Nrf2, as evidenced by its nuclear translocation, increased expression of HO-1 protein, and enhanced Nrf2-dependent ARE-reporter activity. These observations are consistent with previous studies which underscore the role of CDDO-Me as a pharmacological activator of Nrf2 and its downstream antioxidant defenses in various diseases (17, 19, 30, 34).

CDDO-Me mitigates LPS-induced oxidative stress, as shown by a reduction in ROS levels in THP-1-derived macrophages pretreated with CDDO-Me. This antioxidant effect aligns with Nrf2 activation, which regulates the expression of antioxidant genes such as HO-1. Excessive oxidative stress is a hallmark of inflammation and contributes to macrophage dysfunction in various pathological conditions. Supporting this, Pei et al. reported that bardoxolone ameliorates LPS-induced acute lung injury by reducing oxidative stress and inflammation through Nrf2 activation (35). Interestingly, Moerland et al. demonstrated that CDDO-Me reduced lung tumor burden in a cancer mouse model in an Nrf2-dependent manner, with macrophage polarization shifting from a tumor-promoting to a tumor-inhibiting phenotype (34).

The anti-inflammatory properties of CDDO-Me were observed under both basal and LPS-stimulated conditions 6 hours after treatment. In untreated macrophages, CDDO-Me reduced the expression of pro-inflammatory cytokines such as IL-1β and TNF-α, while modestly increasing IL-6 expression. Under LPS-stimulated conditions, pretreatment with CDDO-Me significantly downregulated IL-1β, TNF-α, and IL-6 expression. These findings suggest that CDDO-Me not only suppresses basal inflammatory signaling but also attenuates LPS-induced hyperinflammatory responses, highlighting its potential therapeutic applications in inflammatory diseases.

CDDO-Me also influenced macrophage polarization by modulating the expression of M1 and M2 markers under different environmental stimuli. The expression of M1 and M2 markers was assessed 4 days after polarization. In M0 macrophages, CDDO-Me reduced the expression of both M1 marker genes (CCR7, IL-1β) and M2 marker genes (PPARγ, CCL22, IL-10), suggesting a general dampening of polarization. Similarly, in M1-polarized macrophages, CDDO-Me reduced the expression of both M1 marker genes (IL-23, CCR7, IL-6) and M2 marker gene (CCL22). These results indicate that CDDO-Me may promote a more balanced and less inflammatory macrophage phenotype.

Furthermore, we observed that CDDO-Me enhances the bactericidal activity of macrophages against intracellular *S. aureus.* Pretreatment with CDDO-Me significantly reduced the survival of intracellular bacteria in both THP-1-derived macrophages and bronchoalveolar macrophages. However, this effect was absent in Nrf2 knockout mice, indicating that enhanced bactericidal activity is dependent on Nrf2 activation. These observations corroborate our earlier findings, highlighting the critical role of the Nrf2 signaling pathway in macrophage bactericidal function (17). Supporting evidence from Chi et al. shows that baicalein activates Nrf2 by disrupting the Keap1-Nrf2 connection, thereby reducing oxidative stress and suppressing the M1 phenotype of macrophages (36).

Future studies should investigate whether the MAPK pathways are involved in the ability of CDDO-Me to enhance bacterial clearance. We hypothesize that effects of CDDO-Me may stem from its inhibition of LPS- or *S. aureus*-induced inflammation. Our lab has previously shown that Nrf2 activation reduces oxidative stress and inflammation, potentially through the inhibition of *S. aureus*-induced phosphorylation of JNK and p38 MAPK signaling pathways (37). However, this hypothesis requires further investigations.

Several limitations to consider in the use of CDDO-Me as a treatment include the increased risk of heart failure observed in clinical trials of CDDO-Me in patients with type 2 diabetes mellitus and stage 4 chronic kidney disease (38, 39), as well as gender-related differences in oxidation and inflammation, which make males more prone to higher oxidative stress and inflammation, with a weaker response to stimuli (40). Another limitation is the lower incidence of cardiovascular disease in premenopausal women (41). Recently, a phase 3 clinical trial involving patients diagnosed with Alport syndrome has been conducted, and patients exhibited varying degrees of compromised kidney function at baseline, along with sustained improvements in kidney function and preservation of eGFR (42, 43).

Despite these limitations, the simultaneous activation of Nrf2, suppression of inflammation, reduction of oxidative stress, and enhancement of macrophage bactericidal activity position CDDO-Me as a promising therapeutic candidate for diseases characterized by oxidative stress, inflammation, and immune dysfunction. This is particularly relevant given the increased risk of *S. aureus* infections in patients with CKD, especially those undergoing dialysis, who are highly susceptible to bacterial infections. Future studies should explore its effects *in vivo* in models of chronic inflammatory diseases and infections. Additionally, elucidating the molecular mechanisms underlying its dual effects on macrophage polarization could provide deeper insights into its immunomodulatory properties.

## Data Availability

The original contributions presented in the study are included in the article/supplementary material. Further inquiries can be directed to the corresponding author.

## Glossary

CCL22: C-C Motif Chemokine Ligand 22
CCR7: C-C motif chemokine receptor 7
CDDO-Me: bardoxolone methyl
cDNA: complementary deoxyribonucleic acid
CFU: colony forming unit
DAPI: 4′,6-diamidino-2-phenylindole
DMSO: dimethyl sulfoxide
GAPDH: Glyceraldehyde-3-phosphate dehydrogenase
HO-1: heme oxygenase-1
IL: interleukin
JAK/STAT: Janus kinase/signal transducers and activators of transcription
LPS: lipopolysaccharide
MAPK: mitogen-activated protein kinases
MOI: multiplicity of infection
MTT: 3-(4,5-dimethylthiazol-2-yl)-2,5-diphenyltetrazolium bromide
NF-kB: Nuclear factor kappa-light-chain-enhancer of activated B
Nrf2: nuclear factor erythroid 2–related factor 2
PBS: phosphate-buffered saline
PI3K/AKT/mTOR: phosphoinositide 3 kinase (PI3K)/Akt/mammalian target of rapamycin
PFA: paraformaldehyde
PMA: phorbol 12-myristate 13-acetate
PPARγ: peroxisome proliferator-activated receptor gamma
RAW 264.7: murine macrophage cell line
RIPA: radioimmunoprecipitation assay buffer
RNA: ribonucleic acid
ROS: reactive oxygen species
RT-qPCR: reverse transcription-quantitative polymerase chain reaction
THP-1: human leukemic cell line
TNF-α: tumor necrosis factor-α
